# Forensic Autosomal Short Tandem Repeats and Their Potential Association With Phenotype

**DOI:** 10.3389/fgene.2020.00884

**Published:** 2020-08-06

**Authors:** Nicole Wyner, Mark Barash, Dennis McNevin

**Affiliations:** ^1^Centre for Forensic Science, School of Mathematical and Physical Sciences, Faculty of Science, University of Technology Sydney, Sydney, NSW, Australia; ^2^Department of Justice Studies, San José State University, San Jose, CA, United States

**Keywords:** short tandem repeat, phenotype, forensic marker, DNA profiling, junk DNA, non-coding STRs

## Abstract

Forensic DNA profiling utilizes autosomal short tandem repeat (STR) markers to establish identity of missing persons, confirm familial relations, and link persons of interest to crime scenes. It is a widely accepted notion that genetic markers used in forensic applications are not predictive of phenotype. At present, there has been no demonstration of forensic STR variants directly causing or predicting disease. Such a demonstration would have many legal and ethical implications. For example, is there a duty to inform a DNA donor if a medical condition is discovered during routine analysis of their sample? In this review, we evaluate the possibility that forensic STRs could provide information beyond mere identity. An extensive search of the literature returned 107 articles associating a forensic STR with a trait. A total of 57 of these studies met our inclusion criteria: a reported link between a STR-inclusive gene and a phenotype and a statistical analysis reporting a *p*-value less than 0.05. A total of 50 unique traits were associated with the 24 markers included in the 57 studies. TH01 had the greatest number of associations with 27 traits reportedly linked to 40 different genotypes. Five of the articles associated TH01 with schizophrenia. None of the associations found were independently causative or predictive of disease. Regardless, the likelihood of identifying significant associations is increasing as the function of non-coding STRs in gene expression is steadily revealed. It is recommended that regular reviews take place in order to remain aware of future studies that identify a functional role for any forensic STRs.

## Introduction

Short tandem repeats (STRs) are short repeated sequences of DNA (2–6 bp) that account for approximately 3% of the human genome (Lander et al., 2001). The number of repeat units is highly variable among individuals, which offers a high power of discrimination when analyzed for identification purposes. It is a widely accepted notion that STRs are non-coding in nature and are therefore not implicated in gene expression (Tautz and Schlotterer, 1994; Ramel, 1997; Butler, 2006; Biscotti et al., 2015). There is increasing evidence, however, that non-coding DNA sequences such as STRs may be involved in gene regulation via various mechanisms, hence being associated with phenotype (Sawaya et al., 2013; Chen et al., 2016).

The first STR markers used in forensic casework were selected in 1994 by the Forensic Science Service (FSS) in the United Kingdom for a quadruplex amplification system consisting of four tetranucleotide STRs—TH01, vWA, FES/FPS, and F13A1 (Kimpton et al., 1994). These markers were deemed suitable for PCR amplification due to their simple repeat sequences and their propensity to display regularly spaced alleles differing by four bases; however, the quadruplex system did not offer a high level of discrimination. In 1997, the Federal Bureau of Investigation (FBI) nominated 13 autosomal STR loci to form the core of the Combined DNA Index System (CODIS), a database consisting of profiles contributed by federal, state, and local forensic laboratories. Two of the markers initially selected by the FSS (vWA and TH01) were included within the core CODIS set, whereas FES/FPS and F13A01 were eventually discarded due to low levels of polymorphism. The core set was reviewed in 2010 with an additional seven STRs being implemented from January 1, 2017. The majority of commercially available DNA profiling kits are manufactured to include the core CODIS STR loci (Butler, 2006). In accordance with the DNA Identification Act of 1994, CODIS is bound by stringent privacy protection protocols, in that the stored DNA samples and subsequent analyses be used strictly for law enforcement identification purposes. The DNA Analysis Backlog Elimination Act of 2000 reaffirms that the markers used for forensic applications were specifically selected because they are not known to be associated with any known physical traits or medical characteristics.

The markers nominated for CODIS were specifically chosen due to their location within non-coding regions of the genome; however, claims that non-coding regions play no functional role have been contested in recent years (Cole, 2007; Kaye, 2007; Sarkar and Adshead, 2010). There is increasing evidence that there may be associations between certain STR alleles and medical conditions (von Wurmb-Schwark et al., 2011; Meraz-Rios et al., 2014). This should not be confused with situations where alleles or loci are diagnostic for medical conditions (e.g., trisomy). Additionally, the ability to infer biogeographical ancestry (BGA) from forensic STRs is possible (Graydon et al., 2009; Algee-Hewitt et al., 2016) with investigators using population-specific STR data as intelligence to guide enquiries (Lowe et al., 2001). BGA is correlated with some phenotypes such as blue eye color in Europeans (Gettings et al., 2014) and lighter skin color with increasing distance from the equator (Relethford, 1997). However, the STR genotype *per se* is not causative of BGA phenotype in any direct sense and is mostly associated with BGA as a result of genetic drift (as STRs for forensic use have been selected to exhibit Hardy Weinberg equilibrium). In the event that any CODIS markers are in future found to be linked to a medical condition or physical trait, the analysis of the DNA sample must still be used only for identification purposes pursuant to the DNA Identification Act of 1994.

Katsanis and Wagner (2013) assessed 24 CODIS loci for phenotypic associations, but found no evidence to support the disclosure of any biomedically relevant information. For example, despite the fact that the locus TH01 was associated with as many as 18 traits: from alcoholism to spinocerebellar ataxia, the authors state that association with these traits does not necessarily imply that individual genotypes are causative or predictive of a particular trait. Following this, a statement issued by the Scientific Working Group of DNA Analysis Methods [SWGDAM] (2013) restated that although alternate discoveries may be made in the future, current understanding is that the CODIS loci do not reveal any information beyond identity. There has only been one STR to date that has been removed from consideration as a marker used in human identity testing (Szibor et al., 2005). The STR locus HumARA is located within a coding region on the X-chromosome and has been linked to muscular dystrophy. HumARA is a trinucleotide repeat and these are known to be more prone to disease-causing expansions than tetranucleotide repeats (Orr and Zoghbi, 2007; Castel et al., 2010; Hannan, 2018).

## Materials and Methods

A systematic search of the literature was conducted across three databases (Web of Science, PubMed, and Google Scholar) between August and December 2018. Population data studies, allele frequency studies, validation studies, technique developments, single case reports, mutation analyses, off-ladder allele identification, loss of heterozygosity studies, and locus characterizations were excluded. Additional papers were located by back referencing relevant or similar studies. Following the literature search, each STR was analyzed in the University of California Santa Cruz (UCSC) Genome Browser (Human GRCh38/hg38 Assembly) using the following tracks: Mapping and Sequencing—Base Position-dense; STS Markers-full, Gene and Gene Prediction—GENCODE v29-full; NCBI RefSeq-pack, Phenotype and Literature—OMIM Alleles-full; OMIM Pheno Loci-full; OMIM Genes-full; HGMD Variants-full; GWAS Catalog-full, Regulation—ENCODE Regulation-show; RefSeq Func Elems-full, Variation—Common SNPs(151)-full; FlaggedSNPs(151)-full, Repeats—Microsatellite-full; Simple Repeats-full. The STRs investigated included the 20 CODIS core loci used by the FBI, three extra loci currently used in Australia (Penta E, Penta D, D6S1043), and SE33 which is a core STR in the German national database and has subsequently been incorporated into several European kits.

## Results and Discussion

A total of 57 association studies sourced from three databases met our inclusion criteria: a reported link between a STR-inclusive gene and a phenotype and a statistical analysis reporting a *p*-value less than 0.05. Fifty unique traits were identified across the 24 markers (Supplementary Table 1). Schizophrenia was the trait most frequently described with a total of 11 studies reporting data on 14 different polymorphisms potentially associated with eight loci. Two separate articles investigated the allelic frequency amongst people who attempted suicide and reported a significantly higher frequency amongst 10 different alleles of seven forensic loci. The intronic STR TH01 had the greatest number of studies with 26 reports describing 27 traits potentially linked to 40 different genotypes. Five of these studies were investigating a link to schizophrenia, reporting five polymorphisms that are possibly associated with the disease. No studies associating alleles or genotypes with phenotype were found for Penta E, Penta D, D3S1358, SE33, or D10S1248; however, one study by Shi et al. (2012) investigated the method of diagnosing Down syndrome by testing for a trisomy at the Penta D locus as it is located on chromosome 21. Similarly, six of the 10 articles included for D21S11 were investigating the marker’s efficiency in genetic tests for Down syndrome.

Of the 57 articles proposing an association between a forensic STR and a phenotype, none of them confirmed any particular genotype to be solely causative of a phenotype. Despite 13 of the STRs being located within a functional gene, there were no entries in the Online Mendelian Inheritance in Man (OMIM) database relating any STR-inclusive regions of these genes with a disease. A stand-out result is the number of studies reporting an association between a phenotype with polymorphisms at the TH01 locus.

### TH01

TH01 is located within the first intron of the tyrosine hydroxylase (TH) gene and is commonly characterized by the repeat motif [AATG]_*n*_ or alternatively by the [TCAT]_*n*_ motif, according to GenBank top strand nomenclature. TH is the rate-limiting enzyme involved in the biosynthesis of the catecholamines dopamine, epinephrine, and norepinephrine. Catecholamines act as both neurotransmitters and hormones that assist in maintaining homeostasis (Eisenhofer et al., 2004). As such, a strong relationship has been reported in the literature (Eisenhofer et al., 2004; Ng et al., 2015) between variations in the expression of TH and the development of neurological, psychiatric, and cardiovascular diseases.

Previous studies (McEwen, 2002; Antoni et al., 2006; Bastos et al., 2018) have shown that increased levels of epinephrine and norepinephrine are expressed in individuals experiencing acute or chronic stress. Wei et al. (1997) found that individuals carrying the TH01-9 allele showed the highest levels of serum norepinephrine amongst a population of unrelated healthy adults, whereas carriers of the TH01-7 allele showed the lowest. Barbeau et al. (2003) investigated the relationship between the number of TH01 repeats and hemodynamic parameters in subjects at rest and in response to applied stressors. The results of this study indicate that the 6 and 9.3 TH01 alleles are associated with a decrease in the hemodynamic responses to stress, offering a protective effect to individuals carrying those alleles. Carriers of the TH01-6 allele displayed a lower heart rate reactivity when exposed to stressors with increasing age than those without the TH01-6 allele. Furthermore, individuals carrying TH01-9.3 showed no increase in systolic blood pressure in response to stress, whereas those not possessing the TH01-9.3 allele demonstrated a significant increase in systolic blood pressure reactivity with increasing age. Conversely, the TH01-7 allele was found to be detrimental to blood pressure in those with a greater body mass index (BMI). Subjects carrying TH01-7 displayed a higher resting systolic blood pressure as BMI increases and increased heart rate reactivity in response to stressors with increasing BMI.

TH01-7 was also reported to be significantly more prevalent in patients prone to depression (Chiba et al., 2000). The TH01-8 allele was found more frequently in suicide attempters (Persson et al., 1997), individuals with depression (Serretti et al., 1998), and individuals with delusional disorder (Morimoto et al., 2002). Persson et al. (2000) investigated the influence of the number of TH01 repeats on 30 personality dimensions. Subjects possessing the TH01-8 allele scored higher in the neuroticism facets with significant differences observed between individuals displaying anger, hostility and vulnerability (Persson et al., 2000), compared to non-TH01-8 allele carriers. Nine repeats at the TH01 locus were associated with delusional disorder (Morimoto et al., 2002) and extraversion (Tochigi et al., 2006). Furthermore, Yang et al. (2011) conducted a number of association studies in China and reported that the frequency of TH01-9.3 was higher in those displaying suicidal behavior, and TH01-10 was significantly overrepresented in individuals demonstrating violent behavior including sexual assaults (Yang et al., 2010) and in males with impulsive violent behavior (Yang et al., 2013). TH01 was also linked to various disease states such as schizophrenia (Jacewicz et al., 2006b), predisposition to malaria (Gaikwad et al., 2005; Alam et al., 2011), sudden infant death syndrome (SIDS) (Klintschar et al., 2008; Courts and Madea, 2011), and Parkinson’s disease (Sutherland et al., 2008).

As previously mentioned, TH catalyzes the conversion of tyrosine to levodopa (L-DOPA) which is then converted to dopamine. Dopamine can be further converted into norepinephrine and epinephrine. *In vitro* experiments have previously demonstrated that TH01 can regulate TH gene transcription, displaying a quantitative silencing effect (Albanèse et al., 2001). TH01 alleles inhibited transcription proportionally to the number of repeats. Given that so many vital functions rely on the presence of dopamine and its metabolites (Wei et al., 1997; Meiser et al., 2013), malfunctions of dopaminergic pathways have been associated with the development of numerous psychological diseases (Meiser et al., 2013), and in this review, TH01 was largely connected with schizophrenia (Kurumaji et al., 2001) and Parkinson’s disease (Meiser et al., 2013). The longer TH01-9.3 and TH01-10 alleles, predicted to yield less dopamine, were found more frequently in individuals displaying traits indicative of dopaminergic dysfunction such as impulsive violent behavior (Yang et al., 2013), sexual assault (Yang et al., 2010), and addiction (Sander et al., 1998; Anney et al., 2004).

Some contradictory associations were observed between TH01 and certain phenotypes. For instance, De Benedictis et al. (1998) reported a significant association of >9 TH01 repeats with longevity in male Italian centenarians. Contrariwise, von Wurmb-Schwark et al. (2011) were unable to replicate this result when using the same study design on a German population, just as Bediaga et al. (2015) were also unable to confirm an association in a northern Spanish population. Similarly, there are conflicting reports on the association of TH01-9.3 with SIDS across European populations. In 2008, Klintschar et al. (2008) found that the frequency of the TH01-9.3 allele was significantly higher in SIDS patients than in controls in a German population. This association was further confirmed by Courts and Madea (2011). On the contrary, Studer et al. (2014) were unable to replicate this result in a Swiss population. Further population-based association studies are needed to confirm the existence of associations between TH01 and these phenotypes.

None of the studies investigating TH01 have identified any of the associated genotypes as being causative of disease; therefore, the associations mentioned should only be considered as possible or potential. Many of the traits reported to be associated with TH01 are multifactorial, meaning they are affected by both genes and the environment, such as in the case of Parkinson’s disease (Meiser et al., 2013) and schizophrenia (Zhuo et al., 2019).

### Potential Associations of Other STR Markers

Schizophrenia is a complex heritable mental health disorder characterized by delusions, hallucinations, and impaired social cognition. It is understood that schizophrenia is polygenic with disease burdening alleles being distributed across multiple loci (Giusti-Rodríguez and Sullivan, 2013; Zhuo et al., 2019). Consistent with this notion, our study revealed that schizophrenia was associated with the greatest number of STRs: FGA, TH01, vWA, D2S441, D2S1338, D8S1179, D16S539, and D18S51. One study (Jacewicz et al., 2006a) found that longer repeats in D18S51 and D2S1338 were significantly more frequent in patients than in controls. This trend is consistent with the expansion of trinucleotide repeats in other major psychiatric disorders. Although the inherent complexity of the disease has posed a challenge to researchers, neurotransmitter abnormalities have long been acknowledged as a major contributing factor in the pathogenesis of schizophrenia (Mäki et al., 2005; Modai and Shomron, 2016).

Genetic mutations alone are not enough to trigger the onset and development of schizophrenia; therefore, further research is required in order to explore how genetic risk factors interact with environmental risk factors in the development, onset, and progression of the condition.

Venous thromboembolism (VTE) is a disorder defined by the occurrence of deep vein thrombosis and/or pulmonary embolism. vWF is a glycoprotein that plays a role in platelet adhesion during coagulation; therefore, it is understood that alterations in serum levels of vWF can contribute to thrombosis disorders (Laird et al., 2007). Meraz-Rios et al. (2014) found that vWA-18, TPOX-9, and TPOX-12 were observed more frequently in individuals with venous thrombosis in the Mexican mestizo population. Furthermore, vWA and TPOX have been associated with chronic myeloid leukemia (Wang et al., 2012).

### Trisomys

Down syndrome, or Trisomy-21, can be diagnosed by the presence of a third allele at chromosome 21. This trisomy can be present at any polymorphic marker found on chromosome 21, and there are several studies evaluating the use of D21S11 and Penta D as effective markers in Down syndrome detection (Yoon et al., 2002; Liou et al., 2004; Shi et al., 2012; Guan et al., 2013). Similarly, D18S51 and D13S317 can be used as genetic markers to diagnose the presence of Edwards syndrome (Trisomy-18) and Patau syndrome (Trisomy-13), respectively. Trisomys are an example of a causal association as all individuals with three chromosomes will be affected. While the presence of an extra allele at chromosomes 13, 18, or 21 does not reveal a medical condition unknown to the donor, it does provide additional identifiable information to investigators.

### Cancer

Forensic STRs have been used as genetic markers in several studies to screen for cancer-related alleles. Hui et al. (2014) found that two pairs of alleles (D8S1179-16 with D5S818-13 and D2S1338-23 with D6S1043-11) were found more frequently in gastric cancer patients. Furthermore, a study from China identified a significant association between homozygous alleles at D6S1043 and an increased risk of invasive cervical cancer (Wu et al., 2008). Loss of heterozygosity (LOH) is a genetic mutation that results in the loss of one copy of a heterozygous gene, often resulting in cancer due to loss of functional tumor suppressor genes. LOH in different cancer tissues have been observed at a number of forensic loci such as CSF1PO, FGA, vWA, D3S1358, D5S818, D8S1179, D13S317, and D18S51 in patients with laryngeal cancer (Rogowski et al., 2004). LOH may alter the results of a DNA profile and should be taken into consideration in cases where only cancerous tissue is available for analysis (Peloso et al., 2003; Zhou et al., 2017).

Qi et al. (2018) conducted a study investigating the possibility of using genetic markers rather than related genes to screen for predisposition to lung and liver cancer. This study used CODIS markers to examine the theory of programmed onset which hypothesizes that the occurrence of a chronic disease is independent of age and may instead depend on a programmed onset pattern. The results showed a significant difference in the occurrence of lung cancer between those who carried the D18S51-20 allele and those who did not, and the incidence of liver cancer between those carrying D21S11-30.2 and D6S1043-18 alleles and those who did not. While these results demonstrate CODIS markers being used to predict an individual’s predisposition to cancer, there are an extensive number of cancer-related genes in the genome; therefore, the risk of breaching genetic privacy with this information remains low.

### Y and X STRs

The Y chromosome has accumulated male advantage and fertility genes (Lahn and Page, 1997; Graves, 2006) and so it is possible that phenotypes associated with maleness are associated with Y STRs. X-linked phenotypes (as a result of recessive genes on the X chromosome) are more prevalent in males (because there is no dominant Y chromosome homolog) so there may also be associations with X STRs. In fact, X-linked genes have recently been shown to influence male fertility and sex ratio of offspring in mice (Kruger et al., 2019).

### Association Versus Causation

The association of a STR with a trait or disease does not infer causation. Moreover, some alleles seem to have opposite effects: TH01 allele 9.3 may help with stress (Zhang et al., 2004) but also has a potential link with suicide (Persson et al., 1997; Yang et al., 2011). A genetic variant is considered causative when it is known that the presence of the variant will produce an effect that in turn causes disease (Hu et al., 2018). None of the associations reported in this study offer proof of causation (except for trisomys), rather they propose a general relationship between some STRs used in forensic applications and a phenotype. These relationships may also be explained by confounding variables, bias, or by chance in cases where a significant finding is unable to be replicated by another study. In fact, this review could be seen as a reflection of the broader so-called “replication crisis” in science (Schooler, 2014). Many of the studies reported in this review may not have sufficiently mitigated against the “multiple comparison problem” where a number of comparisons will be significant by chance. By setting our *p*-value threshold to 0.05, we run the risk that 5% of significant results are significant by chance.

Many of the traits that can be predicted by genetic analysis are the result of epistatic interactions between genes and environmental factors. When considering the associations in this review, it is not reasonable to suggest that an individual possessing the more frequently observed allele associated with a trait will express a specific phenotype. There are many underlying mechanisms involved in the development of complex diseases and while the risk of forensic STRs being found to expose revealing medical information is minimal, the presence of a particular allele may indicate heightened potential or risk for a phenotype.

### Molecular Mechanisms

While it remains true that forensic markers are located within non-coding regions, there is growing evidence that STRs in introns and up- or down-stream of genes may affect phenotype. STR mutations in the 5′ untranslated region (UTR) are known to modify gene expression, probably because they serve as protein binding sites (Li et al., 2004). Mutations in the 3′ UTR result in extended mRNA which can be toxic to the cell (Li et al., 2004; La Spada and Taylor, 2010). There are 13 CODIS STRs located in introns (Supplementary Table 2). Mutations in introns can affect mRNA splicing which can result in gene silencing or loss of function (Li et al., 2004; La Spada and Taylor, 2010). The TCAT repeat in the first intron of TH01 acts as a transcription regulatory element *in vitro* (Meloni et al., 1998). Albanèse et al. (2001) reported a reduction in transcriptional activity of TH as the TCAT repeat number varied from three to eight. STRs are also found at high density in promoter regions and it is highly likely that some are implicated in gene expression by modulating spacing of regulatory elements (Gemayel et al., 2012; Sawaya et al., 2013; Gymrek et al., 2016; Quilez et al., 2016; Gymrek, 2017).

There is now etiological support for STRs as causative agents for disease in that they are quite plausibly epigenetic regulators for gene expression when located in introns or up- or down-stream of genes. This may increase prior support for the hypotheses of association and thus reduce the required significance level, as described by Kidd (1993), which is a counter to the “multiple comparison problem” discussed earlier.

## Conclusion

While the results of this study did indicate a large number of phenotypic traits associated with forensic STRs, none were found to be independently causative or predictive of disease. Nevertheless, as there are numerous reported instances of tetranucleotide repeats being implicated in disease and molecular mechanisms have been demonstrated, there remains a strong chance that this inference may change in the near future. One limitation of this study was the sole use of the UCSC genome browser. Future studies may benefit from using a wider range of resources and investigating additional markers such as SNPs in flanking regions, mtDNA and Y-STRs. In the event that a statistically significant association, causal or predictive relationship is discovered, it is not necessarily a valid cause for removal from STR panels, but additional protective measures, such as tightening legislation surrounding genetic privacy, may need to be considered to prevent abuse of this information.

## Author Contributions

NW designed the study, performed the literature review, and wrote the manuscript. MB conceived the project, designed the study, and reviewed and edited the manuscript. DM conceived and managed the project, designed the study, and reviewed and edited the manuscript. All authors contributed to the article and approved the submitted version.

## Conflict of Interest

The authors declare that the research was conducted in the absence of any commercial or financial relationships that could be construed as a potential conflict of interest.
