# Supplementary material for: Forensic Autosomal Short Tandem Repeats and Their Potential Association With Phenotype
Source: Front Genet. 2020 Aug 6;11:884. doi: 10.3389/fgene.2020.00884 (PMC7425049; doi:10.3389/fgene.2020.00884)
Supplement: Supplementary file 1 [file Table_1.DOCX]

| Marker | Location | Intron | Gene Name | Nearest Gene | dbSNPs | Reported Associations | | | | |
| --- | --- | --- | --- | --- | --- | --- | --- | --- | --- | --- |
|  |  |  |  |  |  | Disease caused by gene mutation (OMIM) | Trait | Alleles | p-value | References |
| TPOX | 2p25.3 | 10 | Thyroid peroxidase | - | rs59667255 | Thyroid dyshormonogenesis 2A | Venous Thrombosis | 9, 12 | ≤ 0.0001 | (Meraz-Rios et al., 2014) |
|  |  |  |  |  |  |  | Male impulsive violent behaviour | 8  9  11 | 0.0005  0.0072  <0.0001 | (Yang et al., 2013a) |
|  |  |  |  |  |  |  | Crime of rape | 8, 12 | <0.05 | (Yang et al., 2010) |
|  |  |  |  |  |  |  | Chronic Myeloid Leukaemia | - | <0.05 | (Wang et al., 2012) |
| FGA | 4q31.3 | 3 | Fibrinogen alpha chain | - | - | Afibrinogenemia - congenital  Amyloidosis – familial visceral  Dysfibrinogenemia - congenital  Hypodysfibrinogenemia - congenital | Suicide | 18, 19, 23 | 0.05 | (Yang et al., 2011) |
|  |  |  |  |  |  |  | Chronic obstructive pulmonary disease susceptibility | 20.2 | <0.05 | (Jing-hui et al., 2011) |
|  |  |  |  |  |  |  | Chronic obstructive pulmonary disease protection | 26 | <0.05 | (Jing-hui et al., 2011) |
|  |  |  |  |  |  |  | Schizophrenia | 18 | 0.0354 | (Liu et al., 2004) |
|  |  |  |  |  |  |  | Crime of rape | 22, 23 | <0.05 | (Yang et al., 2010) |
| CSF1PO | 5q32 | 6 | Colony stimulating factor 1 receptor (CSF1R) | - | rs59302630 | Leukoencephalopathy, hereditary diffuse, with spheroids | Male impulsive violent behaviour | 14 | 0.0035 | (Yang et al., 2013a) |
|  |  |  |  |  |  |  | Suicide | 13 | 0.05 | (Yang et al., 2011) |
|  |  |  |  |  |  |  | Chronic Myeloid Leukaemia | - | <0.05 | (Wang et al., 2012) |
| TH01 | 11p15.5 | 1 | Tyrosine hydroxylase | - | rs375879846  rs554658416  rs79373318 | Segawa syndrome, recessive | Schizophrenia | 7  7  7-6  7-6  9.3  9-6  10 | 0.002  0.003  0.031  0.041  0.028  0.0236  0.02 | (Jacewicz et al., 2006b)  (Jacewicz et al., 2006a) (Jacewicz et al., 2008)  (Jacewicz et al., 2006b)  (Jacewicz et al., 2008)  (Jacewicz et al., 2006a)  (Kurumaji et al., 2001)  (Thibaut et al., 1997) |
|  |  |  |  |  |  |  | Malaria | 6, 9 | <0.01 | (Gaikwad et al., 2005; Alam et al., 2011) |
|  |  |  |  |  |  |  | Sudden infant death syndrome | 9.3 | 0.006  0.038 | (Courts and Madea, 2011)  (Klintschar et al., 2008) |
|  |  |  |  |  |  |  | Male impulsive violent behaviour | 10 | <0.0001 | (Yang et al., 2013a) |
|  |  |  |  |  |  |  | Suicide | 8  9.3 | 0.0023  0.046 | (Persson et al., 1997)  (Yang et al., 2011) |
|  |  |  |  |  |  |  | Crime of rape | 10 | <0.05 | (Yang et al., 2010) |
|  |  |  |  |  |  |  | Increased weight, head circumference and length at birth | 10 | <0.05 | (Osada et al., 2007) |
|  |  |  |  |  |  |  | Parkinson’s disease | 9.3 | 0.001 | (Sutherland et al., 2008) |
|  |  |  |  |  |  |  | Depression | 7  8 | <0.0043  0.0001 | (Chiba et al., 2000)  (Serretti et al., 1998) |
|  |  |  |  |  |  |  | Delusional disorder | 8, 9 | <0.02 | (Morimoto et al., 2002) |
|  |  |  |  |  |  |  | High resting systolic blood pressure and increased hemodynamic response to stress | 7 | ≤0.02 | (Barbeau et al., 2003) |
|  |  |  |  |  |  |  | Decreased hemodynamic response to stress | 6  9.3 | ≤0.003  ≤0.002 | (Barbeau et al., 2003) |
|  |  |  |  |  |  |  | Elevated serum noradrenaline levels | 9 | <0.01 | (Wei et al., 1997) |
|  |  |  |  |  |  |  | Low serum noradrenaline levels | 7 | <0.01 | (Wei et al., 1997) |
|  |  |  |  |  |  |  | Elevated serum homovanillic acid levels | 9.3 | <0.05 | (Wei et al., 1997) |
|  |  |  |  |  |  |  | Alcohol-withdrawal delirium | 10 | 0.03 | (Sander et al., 1998) |
|  |  |  |  |  |  |  | Hypertension | 9.3, 10 | <0.0005 | (Sharma et al., 1998) |
|  |  |  |  |  |  |  | Angry hostility | 8 | 0.008 | (Persson et al., 2000) |
|  |  |  |  |  |  |  | Vulnerability | 8 | 0.021 | (Persson et al., 2000) |
|  |  |  |  |  |  |  | Deliberation | 6-10 | 0.035 | (Persson et al., 2000) |
|  |  |  |  |  |  |  | Dutifulness | 6-10 | 0.014 | (Persson et al., 2000) |
|  |  |  |  |  |  |  | Extraversion | 9 | 0.005 | (Tochigi et al., 2006) |
|  |  |  |  |  |  |  | Basal pulse interval | 6  9.3  6-9.3 | 0.007  0.0349  0.0472 | (Zhang et al., 2004) |
|  |  |  |  |  |  |  | Basal heart rate | 6  6-9.3 | 0.0003  0.0305 | (Zhang et al., 2004) |
|  |  |  |  |  |  |  | Post-stress heart rate | 6  6-9.3 | 0.0001  0.0011 | (Zhang et al., 2004) |
|  |  |  |  |  |  |  | Basal plasma epinephrine | 9.3 | 0.0133 | (Zhang et al., 2004) |
|  |  |  |  |  |  |  | Renal norepinephrine excretion | 9.3 | 0.0309 | (Zhang et al., 2004) |
| vWA | 12p13.31 | 40 | von Willebrand factor | - | rs75219269  rs74980505  rs216871  rs145242933 | von Willebrand disease, types 1, 2A, 2B, 2M, 2N and 3 | Intracerebral haemorrhage | 17 combined with  D13S317-11 | 0.021 | (Gai et al., 2016) |
|  |  |  |  |  |  |  | Suicide | 16 | 0.05 | (Yang et al., 2011) |
|  |  |  |  |  |  |  | Schizophrenia | 33.28  14  14  17 | 0.028  0.05  0.014  0.043 | (Liu et al., 2007)  (Yang et al., 2014b)  (Liu et al., 2005)  (Liu et al., 2005) |
|  |  |  |  |  |  |  | Sensitivity to arctigenin | 20 | <0.05 | (Susanti et al., 2013) |
|  |  |  |  |  |  |  | Venous thrombosis | 18 | 0.0232 | (Meraz-Rios et al., 2014) |
|  |  |  |  |  |  |  | Chronic Myeloid Leukaemia | - | <0.05 | (Wang et al., 2012) |
| Penta E | 15q26.2 | - | - | 45.4kb from spermatogenesis associated 8 | - | - | - | - | - | - |
| Penta D | 21q22.3 | 4 | Heat shock transcription factor 2 binding protein | - | - | - | Schizophrenic patients with aggressive behaviour | 10 | 0.0027 | (Yang et al., 2017) |
|  |  |  |  |  |  |  | Down syndrome | Trisomy | - | (Shi et al., 2012) |
| D1S1656 | 1q42.2 | 6 | Calpain 9 | - | rs4847015  rs141376519  rs78443572 | - | Autism | - | Multipoint NPL 3.06  Two-point NPL 3.21 | (Buxbaum et al., 2004) |
| D2S441 | 2p14 | - | - | 29.1kb from C1D nuclear receptor corepressor | rs74640515  rs3038865  rs200211877  rs13019438 | Parkinson’s disease 3  Fibromatosis, gingival 1 | Schizophrenia | - | 0.0006 | (Coon et al., 1998) |
|  |  |  |  |  |  |  | Longevity | 15 | 0.001 | (Bediaga et al., 2015) |
| D2S1338 | 2q35 | - | - | 4.2kb from tensin 1 | rs74556061  rs369803537  rs6736691  rs9678338  rs6736805  rs62182233  rs139208613 | - | Intracerebral haemorrhage | 18 combined with D7S820-13 | 0.023 | (Gai et al., 2016) |
|  |  |  |  |  |  |  | Gastric cancer | 23 combined with D6S1043-11 | <0.05 | (Hui et al., 2014) |
|  |  |  |  |  |  |  | Longevity | 18 | <0.05 | (An et al., 2014) |
|  |  |  |  |  |  |  | Aggressive behaviour | 16 | <0.01 | (Yang et al., 2014a) |
|  |  |  |  |  |  |  | Suicide | 22, 25 | <0.05 | (Yang et al., 2011) |
|  |  |  |  |  |  |  | Schizophrenia | <26 | <0.009 | (Jacewicz et al., 2006a) |
|  |  |  |  |  |  |  | Chronic obstructive pulmonary disease protection | 25 | <0.05 | (Jing-hui et al., 2011) |
| D3S1358 | 3p21.31 | 20 | Leucyl-tRNA Synthetase 2 | - | rs2624663  rs71325067 | Hydrops, lactic acidosis, and sideroblastic anaemia  Perrault syndrome 4 | - | - | - | - |
| D5S818 | 5q23.2 | - | - | 158kb from casein kinase 1 gamma 3 | rs5871056  rs25768 | - | Gastric cancer | 13 combined with D8S1179-16 | <0.05 | (Hui et al., 2014) |
|  |  |  |  |  |  |  | Malaria | 13 | <0.01 | (Gaikwad et al., 2005) |
| D6S1043 | 6q15 | - | - | 49.7kb from cancer susceptibility 6 | rs2325399 | - | Gastric cancer | 11 combined with D2S1338-23 | <0.05 | (Hui et al., 2014) |
|  |  |  |  |  |  |  | Liver cancer | 18 | 0.029 | (Qi et al., 2018) |
|  |  |  |  |  |  |  | Cervical cancer HPV-16 | 112, 132 | <0.0001 | (Wu et al., 2008) |
| SE33 | 6q15 | - | - | 110kb from cannabinoid receptor 1 | rs9362477  rs536914220  rs148521563  rs541691546 | - | - | - | - | - |
| D7S820 | 7q21.11 | 1 | Semaphorin 3A | - | rs59186128  rs7786079  rs7789995  rs61107978  rs548246474  rs16887642 | Hypogonadotropic hypogonadism 16 with or without anosmia | Intracerebral haemorrhage | 13 combined with D2S1338-18 | 0.023 | (Gai et al., 2016) |
|  |  |  |  |  |  |  | Invasive hydatidiform moles | 10 | <0.05 | (Zhang et al., 2006) |
| D8S1179 | 8q24.13 | 3 | Long intergenic non-protein coding RNA 964 | - | rs111782616  rs542851842 | - | Gastric cancer | 16 combined with D5S818-13 | <0.05 | (Hui et al., 2014) |
|  |  |  |  |  |  |  | Cerebral infarction | Heterozygosity | 0.045 | (Hui et al., 2012) |
|  |  |  |  |  |  |  | Schizophrenia | 14 | 0.05 | (Yang et al., 2009) |
| D10S1248 | 10q26.3 | - | - | 2.3kb from O-6-methylguanine-DNA methyltransferase | rs563636310  rs2246512  rs58339994 | - | - | - | - | - |
| D12S391 | 12p13.2 | - | - | 172kb from MANSC domain containing protein 1 | rs77312049 | - | Longevity | 16  17 | 0.002  0.003 | (Bediaga et al., 2015) |
|  |  |  |  |  |  |  | Chronic obstructive pulmonary disease | 19.3 | <0.05 | (Jing-hui et al., 2011) |
| D13S317 | 13q31.1 | - | - | 920kb from long intergenic non-protein coding RNA 564 | rs73525369  rs9546005  rs202043589 | - | Intracerebral haemorrhage | 11 combined with vWA-17 | 0.021 | (Gai et al., 2016) |
|  |  |  |  |  |  |  | Suicide | 8 | <0.05 | (Yang et al., 2011) |
|  |  |  |  |  |  |  | Patau syndrome | Trisomy |  | (Chishti et al., 2014) |
| D16S539 | 16q24.1 | - | - | 6.7kb from long intergenic non-protein coding RNA 917 | rs1728369  rs11642858 | - | Suicide | 9 | <0.05 | (Yang et al., 2011) |
|  |  |  |  |  |  |  | Schizophrenia | Shorter alleles | <0.015 | (Jacewicz et al., 2006a) |
|  |  |  |  |  |  |  | Invasive hydatidiform moles | 9 | <0.05 | (Zhang et al., 2006) |
| D18S51 | 18q21.33 | 1 | Apoptosis regulator BCL-2 | - | rs535823682  rs79317590 | Leukaemia/lymphoma, B-cell, 2 | Schizophrenia | 20-24 | 0.029 | (Jacewicz et al., 2006a) |
|  |  |  |  |  |  |  | Lung cancer | 20 | 0.037 | (Qi et al., 2018) |
|  |  |  |  |  |  |  | Longevity | 17 | <0.05 | (An et al., 2014) |
|  |  |  |  |  |  |  | Transitional cell carcinoma (TCC) of urinary bladder cancer | - | 0.044 | (Saidi et al., 2015) |
|  |  |  |  |  |  |  | Edwards syndrome | Trisomy |  | (Yoon et al., 2002; Crkvenac-Gornik et al., 2007; Chishti et al., 2014) |
| D19S433 | 19q12 | 1 | URI1, prefoldin like chaperone | - | rs147936416 | - | Initiative aggressive behaviour | 14.2  14-14 | 0.0011  0.0008 | (Yang et al., 2013b) |
|  |  |  |  |  |  |  | Prostate cancer aggressiveness | - | 0.0004 | (Witte et al., 2000) |
|  |  |  |  |  |  |  | Aggressive behaviour | 13-14.2 | 0.0000 | (Yang et al., 2014a) |
| D21S11 | 21q21.1 | - | - | 422kb from MIR548X host gene | rs138937726  rs13049099  rs373749807 | - | Liver cancer | 20.2 | 0.026 | (Qi et al., 2018) |
|  |  |  |  |  |  |  | Coronary heart disease | 28.2 | <0.05 | (Liu et al., 2011) |
|  |  |  |  |  |  |  | Down syndrome | Trisomy |  | (Yoon et al., 2002; Liou et al., 2004; Crkvenac-Gornik et al., 2007; Shi et al., 2012; Guan et al., 2013; Chishti et al., 2014) |
|  |  |  |  |  |  |  | Alzheimer’s Disease | - | 0.009 | (Heston et al., 1991) |
|  |  |  |  |  |  |  | Crime of rape | 28 | <0.05 | (Yang et al., 2010) |
| D22S1045 | 22q12.3 | 4 | Interleukin 2 receptor subunit beta | - | rs58127106 | - | Longevity | 11 | 0.005 | (Bediaga et al., 2015) |

**References**

Alam, S., Ferdous, A., Ali, M.E., Ahmed, A., Naved, A.F., and Akhteruzzaman, S. (2011). Forensic microsatellite TH01 and malaria predisposition. *Dhaka Univ. J. Biol. Sci.* 20(1)**,** 1-6.

An, W., Zhang, L., Gong, B., Ren, S., and Liu, H. (2014). Screening of longevity-associated genes based on a comparison between dead and surviving populations. *Gene* 534(2)**,** 379-382. doi: 10.1016/j.gene.2013.08.070.

Barbeau, P., Litaker, M.S., Jackson, R.W., and Treiber, F.A. (2003). A tyrosine hydroxylase microsatellite and hemodynamic response to stress in a multi-ethnic sample of youth. *Ethn. Dis.* 13(2)**,** 186-192.

Bediaga, N.G., Aznar, J.M., Elcoroaristizabal, X., Alboniga, O., Gomez-Busto, F., Artabe, I.A., et al. (2015). Associations between STR autosomal markers and longevity. *Age* 37(5)**,** 8. doi: 10.1007/s11357-015-9818-5.

Buxbaum, J.D., Silverman, J., Keddache, M., Smith, C.J., Hollander, E., Ramoz, N., et al. (2004). Linkage analysis for autism in a subset families with obsessive-compulsive behaviors: evidence for an autism susceptibility gene on chromosome 1 and further support for susceptibility genes on chromosome 6 and 19. *Mol. Psychiatry.* 9(2)**,** 144-150. doi: 10.1038/sj.mp.4001465.

Chiba, M., Suzuki, S., Hinokio, Y., Hirai, M., Satoh, Y., Tashiro, A., et al. (2000). Tyrosine hydroxylase gene microsatellite polymorphism associated with insulin resistance in depressive disorder. *Metabolism* 49(9)**,** 1145-1149. doi: 10.1053/meta.2000.8611.

Chishti, H.M., Ansar, M., Ajmal, M., and Hameed, A. (2014). Application of short tandem repeat markers in diagnosis of chromosomal aneuploidies and forensic DNA investigation in Pakistan. *Gene* 548(2)**,** 217-222. doi: <https://doi.org/10.1016/j.gene.2014.07.035>.

Coon, H., Myles-Worsley, M., Tiobech, J., Hoff, M., Rosenthal, J., Bennett, P., et al. (1998). Evidence for a chromosome 2p13-14 schizophrenia susceptibility locus in families from Palau, Micronesia. *Mol. Psychiatry.* 3(6)**,** 521-527.

Courts, C., and Madea, B. (2011). Significant association of TH01 allele 9.3 and SIDS. *‎J. Forensic Sci.* 56(2)**,** 415-417. doi: 10.1111/j.1556-4029.2010.01670.x.

Crkvenac-Gornik, K., Grubic, Z., Stingl, K., Muzinic, D., Brkljacic-Kerhin, V., and Begovic, D. (2007). Rapid prenatal diagnosis of numerical aberrations of chromosome 21 and 18 by PCR-STR method. *Collegium Antropologicum* 31(3)**,** 859-862.

Gai, L., Sun, C., Yu, W., and Liu, H. (2016). Screening of intracerebral hemorrhage associated allele combinations at different loci using a novel association analysis. *Gene* 579(1)**,** 1-7. doi: 10.1016/j.gene.2015.12.031.

Gaikwad, S., Ashma, R., Kumar, N., Trivedi, R., and Kashyap, V.K. (2005). Host microsatellite alleles in malaria predisposition? *Malar. J.* 4(1)**,** 5. doi: 10.1186/1475-2875-4-50.

Guan, L., Ren, C., Li, H., Gao, L., Jia, N., and Guan, H. (2013). [Practicality of rapid prenatal screening for Down syndrome with PCR-short tandem repeat method]. *Chinese J. of Med. Gen.* 30(3)**,** 277-282. doi: 10.3760/cma.j.issn.1003-9406.2013.03.006.

Heston, L.L., Orr, H.T., Rich, S.S., and White, J.A. (1991). Linkage of an Alzheimer disease susceptibility locus to markers on human chromosome 21. *Am. J. Med. Genet.* 40(4)**,** 449-453. doi: 10.1002/ajmg.1320400415.

Hui, L., Jun, T., Jing, Y., and Yu, W.J. (2012). Screening of cerebral infarction-related genetic markers using a Cox regression analysis between onset age and heterozygosity at randomly selected short tandem repeat loci. *‎J. Thromb. Haemost.* 33(4)**,** 318-321. doi: 10.1007/s11239-012-0724-8.

Hui, L., Liping, G., Jian, Y., and Laisui, Y. (2014). A new design without control population for identification of gastric cancer-related allele combinations based on interaction of genes. *Gene* 540(1)**,** 32-36. doi: 10.1016/j.gene.2014.02.033.

Jacewicz, R., Babol-Pokora, K., Berent, J., Pepinski, and Szram, S. (2006a). Are tetranucleotide microsatellites implicated in neuropsychiatric diseases? *Int. Congr. Ser.* 1288(1)**,** 783-785. doi: <https://doi.org/10.1016/j.ics.2005.09.101>.

Jacewicz, R., Galecki, P., Florkowski, A., and Berent, J. (2008). [Association of the tyrosine hydroxylase gene polymorphism with schizophrenia in the population of central Poland]. *Psychiatr. Pol* 42(4)**,** 583-593.

Jacewicz, R., Szram, S., Gałecki, P., and Berent, J. (2006b). Will genetic polymorphism of tetranucleotide sequences help in the diagnostics of major psychiatric disorders? *Forensic Sci. Int.* 162(1)**,** 24-27. doi: <https://doi.org/10.1016/j.forsciint.2006.06.024>.

Jing-hui, C., Hui, L., and Da-yong, H. (2011). The correlation between chronic obstructive pulmonary disease and DNA short tandem repeat. *Clin. J. Medical Officers* 6(1).

Klintschar, M., Reichenpfader, B., and Saternus, K.S. (2008). A functional polymorphism in the tyrosine hydroxylase gene indicates a role of noradrenalinergic signaling in sudden infant death syndrome. *J. Pediatr.* 153(2)**,** 190-193. doi: 10.1016/j.jpeds.2008.02.032.

Kurumaji, A., Kuroda, T., Yamada, K., Yoshikawa, T., and Toru, M. (2001). An association of the polymorphic repeat of tetranucleotide (TCAT) in the first intron of the human tyrosine hydroxylase gene with schizophrenia in a Japanese sample. *J. Neural Transm.* 108(4)**,** 489-495. doi: 10.1007/s007020170069.

Liou, J.D., Chu, D.C., Cheng, P.J., Chang, S.D., Sun, C.F., Wu, Y.C., et al. (2004). Human chromosome 21-specific DNA markers are useful in prenatal detection of Down syndrome. *Ann. Clin. Lab. Sci.* 34(3)**,** 319-323.

Liu, H., Weijian, Y., Fang, F., Wang, X., Yang, G., Liu, B., et al. (2005). Polymorphism of microsatellite DNA vWA in random population and schizophrenic patients. *Chinese J. Tiss. Eng. Res.* 9(24)**,** 252-254.

Liu, H., Ye, J., Mu, R., and Yu, W.J. (2011). Novel association analysis between 9 short tandem repeat loci polymorphisms and coronary heart disease based on a cross-validation design. *Atherosclerosis* 218(1)**,** 151-155. doi: 10.1016/j.atherosclerosis.2011.05.024.

Liu, H., Yu, W., Wang, X., Fang, F., Yang, G., Zhou, J., et al. (2007). Number of STR repeats as a potential new quantitative genetic marker for complex diseases, illustrated by schizophrenia. *Biochem. Genet.* 45(9-10)**,** 683-689. doi: 10.1007/s10528-007-9105-y.

Liu, H., Yu, W.J., Fang, F., Wang, X.B., Liang, X.H., Yang, G., et al. (2004). Correlation of microsatellite DNA FGA gene polymorphism with occurrence and rehabilitation of schizophrenia. *Chin. J. Clin. Rehab.* 8(12)**,** 2286-2287.

Meraz-Rios, M.A., Majluf-Cruz, A., Santana, C., Noris, G., Camacho-Mejorado, R., Acosta-Saavedra, L.C., et al. (2014). Association of vWA and TPOX polymorphisms with venous thrombosis in Mexican mestizos. *BioMed Res. Int.***,** 9. doi: 10.1155/2014/697689.

Morimoto, K., Miyatake, R., Nakamura, M., Watanabe, T., Hirao, T., and Suwaki, H. (2002). Delusional disorder: molecular genetic evidence for dopamine psychosis. *Neuropsychopharmacology* 26(1)**,** 794. doi: 10.1016/S0893-133X(01)00421-3.

Osada, H., Seki, K., and Sekiya, S. (2007). Genetic variations within the insulin gene region are associated with accelerated fetal growth. *Tohoku J. Exp. Med.* 212(1)**,** 27-34. doi: 10.1620/tjem.212.27.

Persson, M.L., Wasserman, D., Geijer, T., Jonsson, E.G., and Terenius, L. (1997). Tyrosine hydroxylase allelic distribution in suicide attempters. *Psychiatry Res* 72(2)**,** 73-80.

Persson, M.L., Wasserman, D., Jonsson, E.G., Bergman, H., Terenius, L., Gyllander, A., et al. (2000). Search for the influence of the tyrosine hydroxylase (TCAT)n repeat polymorphism on personality traits. *Psychiatry Res.* 95(1)**,** 1-8. doi: 10.1016/s0165-1781(00)00160-8.

Qi, X., Yu, Y.J., Ji, N., Ren, S.S., Xu, Y.C., and Liu, H. (2018). Genetic risk analysis for an individual according to the theory of programmed onset, illustrated by lung and liver cancers. *Gene* 673(1)**,** 107-111. doi: 10.1016/j.gene.2018.06.044.

Saidi, S., Popov, Z., Stavridis, S., and Panov, S. (2015). Alterations of microsatellite loci GSN and D18S51 in urinary bladder cancer. *Hippokratia* 19(3)**,** 200-204.

Sander, T., Harms, H., Rommelspacher, H., Hoehe, M., and Schmidt, L.G. (1998). Possible allelic association of a tyrosine hydroxylase polymorphism with vulnerability to alcohol-withdrawal delirium. *Psychiatr. Genet.* 8(1)**,** 13-17.

Serretti, A., Macciardi, F., Verga, M., Cusin, C., Pedrini, S., and Smeraldi, E. (1998). Tyrosine hydroxylase gene associated with depressive symptomatology in mood disorder. *Am. J. Med. Genet.* 81(2)**,** 127-130. doi: 10.1002/(SICI)1096-8628(19980328)81:2<127::AID-AJMG1>3.0.CO;2-T.

Sharma, P., Hingorani, A., Jia, H., Ashby, M., Hopper, R., Clayton, D., et al. (1998). Positive association of tyrosine hydroxylase microsatellite marker to essential hypertension. *Hypertension* 32(4)**,** 676-682. doi: 10.1161/01.HYP.32.4.676.

Shi, Y.F., Li, X.Z., Li, Y., Zhang, X.L., Zhang, Y., and Yue, T.F. (2012). [Diagnosis of Downs syndrome using short tandem repeat loci D21S11, D21S1440 and Penta D]. *Zhonghua Yi Xue Yi Chuan Xue Za Zhi* 29(4)**,** 443-446. doi: 10.3760/cma.j.issn.1003-9406.2012.04.014.

Susanti, S., Iwasaki, H., Inafuku, M., Taira, N., and Oku, H. (2013). Mechanism of arctigenin-mediated specific cytotoxicity against human lung adenocarcinoma cell lines. *Phytomedicine* 21(1)**,** 39-46. doi: <https://doi.org/10.1016/j.phymed.2013.08.003>.

Sutherland, G., Mellick, G., Newman, J., Double, K.L., Stevens, J., Lee, L., et al. (2008). Haplotype analysis of the IGF2-INS-TH gene cluster in Parkinson's disease. *Am. J. Med. Genet. B Neuropsychiatr. Genet.* 147B(4)**,** 495-499. doi: 10.1002/ajmg.b.30633.

Thibaut, F., Ribeyre, J.M., Dourmap, N., Meloni, R., Laurent, C., Campion, D., et al. (1997). Association of DNA polymorphism in the first intron of the tyrosine hydroxylase gene with disturbances of the catecholaminergic system in schizophrenia. *Schizophr. Res.* 23(3)**,** 259-264. doi: 10.1016/s0920-9964(96)00118-1.

Tochigi, M., Otowa, T., Hibino, H., Kato, C., Otani, T., Umekage, T., et al. (2006). Combined analysis of association between personality traits and three functional polymorphisms in the tyrosine hydroxylase, monoamine oxidase A, and catechol-O-methyltransferase genes. *J. Neurosci. Res.* 54(3)**,** 180-185. doi: <https://doi.org/10.1016/j.neures.2005.11.003>.

Wang, Z.L., Dai, L., Li, S., Qiu, G.Q., and Wu, H.Q. (2012). [Comparison of allelic frequencies of 15 short tandem repeat loci between chronic myeloid leukemia patients and non-related healthy individuals]. *Chin. J. Med. Genet.* 29(3)**,** 306-308. doi: 10.3760/cma.j.issn.1003-9406.2012.03.013.

Wei, J., Ramchand, C.N., and Hemmings, G.P. (1997). Possible association of catecholamine turnover with the polymorphic (TCAT)n repeat in the first intron of the human tyrosine hydroxylase gene. *Life Sci.* 61(14)**,** 1341-1347. doi: <https://doi.org/10.1016/S0024-3205(97)00679-6>.

Witte, J.S., Goddard, K.A., Conti, D.V., Elston, R.C., Lin, J., Suarez, B.K., et al. (2000). Genomewide scan for prostate cancer-aggressiveness loci. *Am. J. Hum. Genet.* 67(1)**,** 92-99. doi: 10.1086/302960.

Wu, Y., Zhang, Q., Liu, B., and Yu, G. (2008). The analysis of the entire HLA, partial non-HLA and HPV for Chinese women with cervical cancer. *J. Med. Virol.* 80(10)**,** 1808-1813. doi: 10.1002/jmv.21251.

Yang, C., Ba, H., Gao, Z., Zhao, H., Yu, H., and Guo, W. (2013a). Case-control study of allele frequencies of 15 short tandem repeat loci in males with impulsive violent behavior. *Shanghai Arch. Psychiatry.* 25(6)**,** 354-363. doi: 10.3969/j.issn.1002-0829.2013.06.004.

Yang, C., Ba, H., Tan, X., Zhao, H., Zhang, S., and Yu, H. (2017). [Association of aggressive behaviors of schizophrenia with short tandem repeats loci]. *Zhonghua Yi Xue Yi Chuan Xue Za Zhi* 34(6)**,** 901-904. doi: 10.3760/cma.j.issn.1003-9406.2017.06.027.

Yang, C., Ba, H., Yu, H., Gao, Z., Zhao, H., and Sun, J. (2014a). [Association of 15 short tandem repeats loci with aggressive behavior]. *Chinese J. med. genet.* 31(1)**,** 101-105. doi: 10.3760/cma.j.issn.1003-9406.2014.01.024.

Yang, C., Ba, H., and Zhao, H. (2011). Association study between the genetic polymorphism of 15 STR loci and the suicide behavior in Jiangsu province. *J. Psych.* 1(1)**,** 9.

Yang, C., Huajie, B., Gao, Z., Lin, Z., Zhao, H., Liu, B., et al. (2010). Association study between the genetic polymorphism of 15 STR loci and the crime of rape. *Chin. J. Behavioral Medicine and Brain Science* 19(5)**,** 421-424.

Yang, C., Huajie, B., Haiying, Y., Gao, Z., Zhao, H., and Guo, W. (2013b). Association study of the genetic polymorphism of D2S1338 and D19S433 loci and the initiative-aggressive behavior in male population. *Chinese J. Behav. Med. and Brain Sci.* 1(2)**,** 127-130.

Yang, G., Luo, H., Sun, W., and Liu, H. (2009). Relationship between polymorphism of gene D8S1179, D21S11, D18S51 and schizophrenia. *J. Dalian Med. Univ.* 31(5)**,** 510-512.

Yang, G., Wei, W., and Liu, H. (2014b). Relationship between polymorphism of microsatellite vWA and D5S818 with schizophrenia. *J. Dalian Med. Univ.* 3(1)**,** 8.

Yoon, H.R., Park, Y.S., and Kim, Y.K. (2002). Rapid prenatal detection of Down and Edwards syndromes by fluorescent polymerase chain reaction with short tandem repeat markers. *Yonsei Med. J.* 43(5)**,** 557-566. doi: 10.3349/ymj.2002.43.5.557.

Zhang, L., Rao, F., Wessel, J., Kennedy, B.P., Rana, B.K., Taupenot, L., et al. (2004). Functional allelic heterogeneity and pleiotropy of a repeat polymorphism in tyrosine hydroxylase: prediction of catecholamines and response to stress in twins. *Physiol. Genomics* 19(3)**,** 277-291. doi: 10.1152/physiolgenomics.00151.2004.

Zhang, X.W., Zhu, H.B., Wu, S.Y., Gao, R.L., Han, J.S., Wang, X.Y., et al. (2006). Distribution of the alleles at loci D16S539, D7S820, and D13S317 in hydatidiform mole genome from Chinese women and its relationship with clinical prognosis. *Cancer Genet. Cytogenet.* 164(2)**,** 133-136. doi: <https://doi.org/10.1016/j.cancergencyto.2005.07.017>.
