# Supplementary material for: Forensic Autosomal Short Tandem Repeats and Their Potential Association With Phenotype
Source: Front Genet. 2020 Aug 6;11:884. doi: 10.3389/fgene.2020.00884 (PMC7425049; doi:10.3389/fgene.2020.00884)
Supplement: Supplementary file 2 [file Table_2.DOCX]

Supplementary Table 2. Intragenic STRs and their respective gene function

| STR | Gene | Function |
| --- | --- | --- |
| TPOX | Thyroid peroxidase | Thyroid hormone synthesis |
| FGA | Fibrinogen alpha chain | Component of blood clots |
| CSF1PO | Colony stimulating factor 1 receptor | Production and differentiation of macrophages |
| TH01 | Tyrosine hydroxylase | Catecholamine synthesis |
| vWA | von Willebrand factor | Blood coagulation |
| Penta D | Heat shock transcription factor 2 binding protein | Activate heat-shock response genes under conditions of heat or other stresses. |
| D1S1656 | Calpain 9 | Tumour suppressor |
| D3S1358 | Leucyl-tRNA synthetase 2 | Synthesis of mitochondrial proteins |
| D7S820 | Semophorin 3A | Neuronal pattern development |
| D8S1179 | Long intergenic non-protein coding RNA 964 | Non-coding RNA transcription |
| D18S51 | Apoptosis regulator BCL-2 | Controls mitochondrial membrane permeability |
| D19S433 | URI1 | Scaffolding protein that also plays a role in transcription |
| D22S1045 | Interleukin 2 receptor subunit beta | Assists in receptor mediated endocytosis |
